# Systematic total arch replacement with thoraflex hybrid graft in acute type A aortic dissection: A single centre experience

**DOI:** 10.3389/fcvm.2022.997961

**Published:** 2022-10-14

**Authors:** Pierpaolo Chivasso, Generoso Mastrogiovanni, Vito Domenico Bruno, Mario Miele, Mario Colombino, Donato Triggiani, Francesco Cafarelli, Rocco Leone, Felice Rosapepe, Matteo De Martino, Elvira Morena, Ivana Iesu, Rodolfo Citro, Paolo Masiello, Severino Iesu

**Affiliations:** ^1^Department of Emergency Cardiac Surgery, University Hospital San Giovanni di Dio e Ruggi d'Aragona, Salerno, Italy; ^2^Bristol Medical School, Translational Health Science Department, Bristol, United Kingdom; ^3^Department of Cardiac Anesthesia, University Hospital San Giovanni di Dio e Ruggi d'Aragona, Salerno, Italy; ^4^Department of Cardiology, University Hospital San Giovanni di Dio e Ruggi d'Aragona, Salerno, Italy

**Keywords:** aortic arch surgery, FET, frozen elephant trunk, hybrid arch surgery, acute type A aortic dissection

## Abstract

**Introduction:**

In the last two decades, a more aggressive approach has been encouraged to treat patients with acute type A aortic dissection (ATAAD), extending the repair to the aortic arch and proximal descending thoracic aorta with the frozen elephant trunk (FET) implantation. Here, we report our single-centre experience with the FET technique for the systematic treatment of emergency type A aortic dissection.

**Materials and methods:**

Between December 2017 and January 2022, 69 consecutive patients were admitted with ATAAD; of those, 66 patients (62.9 ± 10.2 years of age, 81.8% men) underwent emergency hybrid aortic arch and FET repair with the multibranched Thoraflex hybrid graft and were enrolled in the study. Primary endpoints were 30 days- and in-hospital mortality. Secondary endpoints were postoperative morbidity and follow-up survival. To better clarify the impact of age on surgical outcomes, we have divided the study population into two groups: group A for patients <70 years of age (47 patients), and group B for patients ≥70 years (19 patients). Time-to-event analysis has been conducted using the Log-rank test and is displayed with Kaplan-Meier curves. A multiple Cox proportional Hazard model was developed to identify predictors of long-term survival with a stepwise backward/forward selection process.

**Results:**

30-days- and in-hospital mortality were 10.6 and 13.6%, respectively. Stroke occurred in three (4.5%) patients. Two (3.0%) patients experienced spinal cord ischemia. We did not find any statistically significant difference between the two groups in terms of main post-operative outcomes. The multivariable Cox proportional hazard model showed left ventricular ejection fraction (HR: 0.83, 95% CI: 0.79–0.92, *p* < 0.01), peripheral vascular disease (HR: 15.8, 95% CI: 3.9–62.9, *p* < 0.01), coronary malperfusion (HR: 0.10, 95% CI: 0.01–0.77, *p* =0.03), lower limbs malperfusion (HR: 5.1, 95% CI: 1.10–23.4, *p* = 0.04), and cardiopulmonary bypass time (HR: 1.02, 95% CI: 1–1.04, *p* = 0.01) as independent predictors of long term mortality.

**Conclusions:**

Frozen elephant trunk repair to treat emergency type A aortic dissection appears to be associated with good early and mid-term clinical outcomes even in the elderly.

## Introduction

Acute type A aortic dissection (ATAAD) is one of the most dangerous and fatal cardiovascular emergencies. Aortic rupture and subsequent cardiac tamponade as well as systemic malperfusion are the most common cause of death as a result of this potentially deadly condition. In most of the cases, emergency surgery is the only therapeutic choice (1, 2). Although substantial improvements in surgical repair techniques and post-operative management have enhanced outcomes for this subset of patients, the largest registries still show high in-hospital mortality of 15–20% (3–6). Traditionally, a less aggressive approach limited to ascending aorta with or without hemiarch replacement has been adopted to reduce the surgical risk (7, 8). However, pathologic remodeling with dilation of the residual dissected aorta and subsequent risk of rupture requiring further intervention in the future may occur. Nowadays, the frozen elephant trunk (FET) technique has become a valuable alternative to treat aortic disease when the arch and the thoracic aorta are involved, both in elective and emergency settings (9–12). Particularly, in acute aortic dissection, the use of FET can lead to expansion and stabilization of the true lumen and can cover eventual additional tears in the stented part of the aortic arch or proximal descending thoracic aorta (DTA) (13, 14). Despite these potential advantages, only a restricted number of institutions have employed this procedure to treat aortic dissection (15–33).

The aim of this study is to review our systematic experience with arch reconstruction and the FET technique for patients presenting with acute type A dissection. A secondary aim is to evaluate the impact of age on the postoperative outcome after this type of surgery.

## Materials and methods

### Study population and definitions

This is a single-center, retrospective, observational study based on prospectively collected data obtained from institutional cardiac surgery dataset at the University Hospital San Giovanni di Dio and Ruggi d'Aragona, Salerno, Italy. The study was conducted in accordance with the principles of the Declaration of Helsinki. Institutional board approval was obtained for the study, and patient consent was waived. Between December 2017 and January 2022, 69 consecutive patients presented at our unit with the diagnosis of ATAAD. The Glasgow Coma Scale was used to assess the level of consciousness, and patients were classified into the following 3 subgroups based on their Glasgow Coma Scale scores: severe, 3–8; moderate, 9–12; and mild, 13–15 (34). We did not perform AAAD repair in patients whose severe coma persisted for more than 10 h. There was only 1 non-operative management patient in this study period. Two patients were treated with a more conservative approach (ascending aorta and hemiarch replacement) due to extremely poor general conditions at presentation. All the other 66 patients underwent repair with the FET technique using Thoraflex hybrid (Terumo Aortic, Scotland) prosthesis and were enrolled in the present study. Fifty-four (81.8%) were male and the mean age was 62.8 (±10.1) years. All the surviving patients were followed-up until May 2022. Patient demographics are summarized in Table 1. To better clarify the impact of age on surgical outcomes, we have divided the study population into two groups: group A for patients <70 years of age (47 patients) and group B for patients ≥70 years (19 patients, Table 1). Emergency surgery was defined as surgery conducted within 24 h of unscheduled admission. Patients were considered to have a chronic obstructive pulmonary disease (COPD) if they had any of the following conditions: long-term use of bronchodilators or steroids for lung disease before admission; outpatient visits including a diagnosis of COPD; and preoperative lung function test with evidence of an obstructive pattern, Patients were considered to have diabetes if they had any of the following conditions: receipt of insulin or oral hypoglycemic medications before admission; outpatient visits including a diagnosis of diabetes mellitus on two occasions; or a previous inpatient stay with a discharge diagnosis of diabetes mellitus. Cardiogenic shock was defined as a preoperative systolic blood pressure <90 mmHg or a cardiac index <2.0 l/min/m2 at arrival. Malperfusion syndromes were defined as symptoms due to disrupted blood flow to the coronary arteries, central nervous system, or peripheral arteries. Any patient with neurological symptoms or syncope that was apparently caused by cardiogenic shock was excluded from the classification of cerebral malperfusion (35).

**Table 1:** Preoperative characteristics.

**Characteristics**	**Overall (66)**	**Group A (47)**	**Group B (19)**	***p*-value**
Age (years)	62.9 (10.2)	58 (7.9)	75 (3)	< 0.001
Male gender	54 (81.8)	41 (87.2)	13 (68.4)	0.089
COPD	13 (19.7)	11 (23.4)	2 (10.5)	0.3
History of hypertension	57 (86.4)	41 (87.2)	16 (84.2)	0.7
CKD	6 (9.1)	6 (12.7)	0	na
History of cancer	3 (4.5)	2 (4.2)	1 (5.3)	>0.9
Diabetes mellitus	7 (10.6)	4 (8.5)	3 (15.7)	0.4
Peripheral vascular disease	8 (12.1)	4 (8.5)	4 (21.0)	0.2
Previous cardiac surgery	3 (4.5)	1 (2.1)	2 (10.5)	0.2
Previous cerebrovascular accident	1 (1.5)	1 (2.1)	0	na
Left ventricle ejection fraction (perc.)	57 (6.3)	57.4 (7.3)	59.1 (4.2)	0.28
Bicuspid aortic valve	1 (1.5)	1 (2.1)	0	na
Marfan syndrome	1 (1.5)	1 (2.1)	0	na
Coronary malperfusion at presentation	10 (15.2)	6 (12.8)	4 (21.0)	0.5
Preoperative hemoglobin level (g/dl)	12.7 (1.7)	13.1 (1.6)	11.9 (1.8)	0.017
eGFR	68.1 (27.5)	67.3 (27.7)	70.1 (27.8)	0.14
Cerebral malperfusion at presentation	8 (12.1)	4 (8.5)	4 (21.0)	0.2
Abdominal malperfusion at presentation	11 (16.7)	9 (19.1)	2 (10.5)	0.5
Lower limb ischaemia at presentation	7 (10.6)	6 (12.8)	1 (5.3)	0.7
**Penn classification**				0.8
Aa	32 (48.5)	23 (48.9)	9 (47.3)	
Ab	21 (31.8)	16 (34.0)	5 (26.3)	
Ac	6 (9.1)	4 (8.5)	2 (10.5)	
Abc	7 (10.6)	4 (8.5)	3 (15.8)	
Preoperative lactate level	1.68 (1.48)	1.62 (1.33)	1.84 (1.8)	0.7

Group A: age at surgery <70 years; group B: age at surgery ≥ 70 years; COPD, Chronic Obstructive Pulmonary Disease; CKD, Chronic Kidney Disease; eGFR: estimated glomerular filtration rate. Data are reported as mean (SD) for numerical variables and as count (%) for categorical variables.

### Thoraflex hybrid FET graft and size selection

The Thoraflex hybrid is a vascular graft designed for complex aortic arch surgery. It consists of a proximal not-stented tubular gelatin-coated Dacron graft and a distal polyester-made stent-graft with a self-expandable nitinol stent, which is deployed anterogradely into the aortic arch/DTA during circulatory arrest. The hybrid prosthesis has four integrated lateral branches: three on the dorsal side for the single reimplantation of supra-aortic vessels and one on the ventral side for systemic perfusion. Between the two portions, there is a sewing collar that makes the distal anastomosis of the prosthesis to the aortic arch wall easier. The Thoraflex hybrid comes in different sizes: the proximal part diameter varies from 22 to 32 mm, and the stented part from 24 to 40 mm. Two different distal lengths are available: 100 and 150 mm. The combination of the varied sizes and lengths allows us to tailorize the graft to the patient's anatomy and pathologic condition. In our series, the decision on stented graft size was figured out by the total aortic diameter and relative diameters of the true and false lumen at the level of the landing zone, based on the exact evaluation of preoperative CT angiogram of the aorta for the entire cohort of patients. No oversizing was performed to reduce the risk of rupture or distal stent graft-induced new entries (DSINEs). To minimize the risk of spinal cord ischemia, we only implanted the 100 mm length stented graft.

### Surgical technique

**Figure 1:**
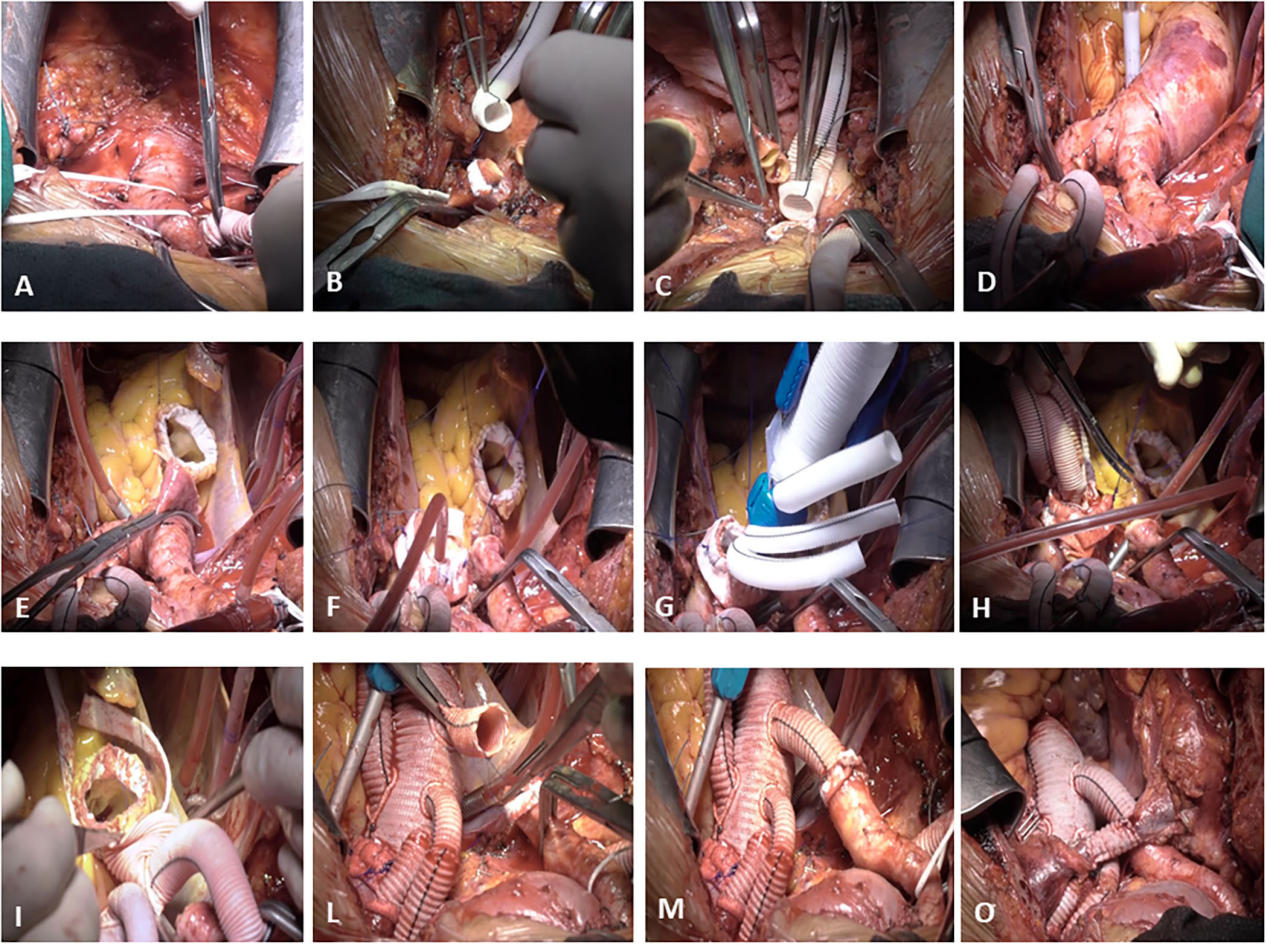
De-branching first surgical technique. The right subclavian artery is isolated from the anterior mediastinum and an end-to-side Dacron vascular graft (8 or 10 mm, depending on the native vessel size) is interposed to avoid direct cannulation of the artery **(A)**. During the first cooling phase, from a beating heart, the left common carotid artery (LCCA) **(B)** and the left subclavian artery (LSA) **(C)** are isolated and prepared for selective cannulation with the interposition of a vascular Dacron graft. After the completion of debranching **(D)**, the ascending aorta is cross-clamped and opened, cardioplegia is administered, and the proximal aortic valve and root reconstruction are performed in different manners as required by the case **(E)**. The brachiocephalic artery is clamped, the aortic arch is then opened and inspected, and the landing zone (usually zone 2) is reinforced with internal and external teflon strips **(F)**. At this stage, the distal stent graft of the Thoraflex hybrid is released into the DTA **(G)**. The strengthened collar of the prosthesis is sutured to the aorta **(H)**, and, after cannulation of the fourth lateral branch and accurate de-airing, a systemic perfusion is resumed. The anastomosis between the hybrid prosthesis and sino-tubular junction (either native or prosthetic, depending on the proximal repair) is then completed **(I)** and the cross-clamp is released. The prosthesis-elongated supra-aortic vessels are then termino-terminally re-anastomosed to the corresponding branches of the Thoraflex hybrid **(L)**, starting with the LSA to LCCA and finally to the brachiocephalic artery **(M)**. Finally, the anonymous vein is reconstructed with the interposition of an 8 mm Dacron vascular graft **(O)**.

Our “debranching first” FET technique (Figure 1) has already been described in earlier reports (36, 37). The usual incision was normally extended in a small bilateral supra-clavicular cervicotomy to improve access to the epiaortic vessels. The right subclavian artery (isolated from anterior mediastinum) or the right axillary artery (isolated in the right sub clavicular region) were routinely employed as the arterial site for central cannulation. In all cases, an end-to-side Dacron vascular graft (8 or 10 mm, depending on the native vessel size) was interposed to avoid direct cannulation of the artery. The right atrium and right superior pulmonary vein were cannulated for venous return and left ventricle venting. A home-made 4-branched perfusion circuit was used for extracorporeal circulation. Cooling to 26, 28°C for hypothermic circulatory arrest was employed in all cases. During the first cooling phase, from a beating heart, the left common carotid artery (LCCA) and the left subclavian artery (LSA) were isolated and prepared for selective cannulation with the interposition of a vascular Dacron graft. The vessel perfusion was sequentially started to achieve complete antegrade “trilateral” cerebral perfusion before clamping the ascending aorta. After debranching completion, at 30°C core temperature, the ascending aorta was cross-clamped and opened and cardioplegia was administered. A single dose of Custodiol^®^ cardioplegia was routinely administered selectively in the coronary ostia. The proximal aortic valve and root reconstruction were performed in different manners as required by the case as shown in Table 2. At a temperature of 26–28°C, the brachiocephalic artery was clamped and the selective antegrade perfusion began at 10–15 ml/kg/min, to stop the systemic circulation. The aortic arch was then opened and inspected. The landing zone (usually zone 2) was reinforced with internal and external teflon strips. At this stage, the distal stent graft of the Thoraflex hybrid was released into the DTA. The strengthened collar of the prosthesis was sutured to the aorta, and, after cannulation of the fourth lateral branch and accurate de-airing, systemic perfusion was resumed, starting to rewarm the body. In the last 19 cases, to reduce lower body ischemia time, a blood flow of around 1,200 ml for spinal and splanchnic perfusion was started through the lateral branch of the Thoraflex hybrid even before completing the collar's anastomosis. The anastomosis between the hybrid prosthesis and sino-tubular junction (either native or prosthetic, depending on the proximal repair) was then completed and the cross-clamp was released. The prosthesis-elongated supra-aortic vessels were then termino-terminally re-anastomosed to the corresponding branches of the Thoraflex hybrid, starting with the LSA to LCCA and finally at the brachiocephalic artery.

**Table 2:** Operative characteristics.

	**Overall (66)**	**Group A (47)**	**Group B (19)**	***p*-value**
CPB time (min)	204.5 (45.2)	205.1 (43.5)	203 (50.3)	0.7
Aortic cross-clamp time (min)	115.1 (36.1)	118 (35.8)	108.1 (36.9)	0.2
HCA time (min)	28.7 (6.9)	30 (6.1)	25.5 (7.9)	0.046
Intraoperative lactate level (peak)	5.7 (2.8)	5.4 (2.2)	6.5 (3.8)	0.6
**Concomitant procedures**				
CABG	11 (16.6)	6 (12.8)	5 (26.3)	0.3
AV replacement	5 (7.6)	4 (8.5)	1(5.3)	0.4
**Aortic root surgery**				
Florida Sleeve	12 (18.2)	9 (19.1)	3 (15.8)	0.9
Modified Bentall	5 (7.6)	3 (6.4)	2 (10.5)	0.6

Group A: age at surgery <70 years; group B: age at surgery ≥ 70 years; CPB, Cardiopulmonary Bypass; HCA, Hypothermic Circulatory Arrest (including selective antegrade cerebral and visceral perfusion); CABG, Coronary Artery Bypass Graft; AV, Aortic Valve. Data are reported as mean (SD) for numerical variables and as count (%) for categorical variables.

### Endpoints

The primary endpoints of the study were 30-days and in-hospital mortality, defined as death due to any cause during the postoperative course at 30 days and until discharge, respectively.

Secondary endpoints included postoperative stroke (defined as clinical and radiological evidence of a new postoperative cerebrovascular event -CVA-), spinal cord injury, return to the operating room for cardiac causes, renal failure requiring replacement therapy, respiratory insufficiency requiring prolonged ventilation and/or tracheostomy, deep sternal wound infection involving sternal bone and/or mediastinal structures, recurrent laryngeal nerve palsy, and in-hospital length of stay.

### Statistical analysis

Data are presented as mean and standard deviation (SD) for continuous numerical variables and as count and percentages for categorical variables. Numerical variables have been compared using the Student *t*-test or Mann-Whitney U test, while categorical variables have been compared with the chi-square test or Fisher exact test as appropriate. Time-to-event analysis has been conducted using the Log-rank test and displayed with Kaplan-Meier curves. A multiple Cox proportional Hazard model was developed to identify predictors of long-term survival with a stepwise backward/forward selection process. The alpha error was set at 0.05 for significance and all tests are two-sided. Missing values were screened before analysis: every variable with more than 5% of the missing value was eliminated from the final dataset. The remaining variables were imputed using simple imputation models. The statistical analysis was conducted with R version 3.6.0 (2019-04-26) (38).

## Results

The mean follow-up was 19.7 ± 17.4 months. The distributions of baseline characteristics for the overall population are presented in Table 1. Three (4.5%) patients had already undergone cardiac surgery. Thirty-six patients (54.5%) were presented with peripheral malperfusion. Particularly, 10 (15.2%) patients were presented with coronary malperfusion, 8 (12.1%) patients with cerebral malperfusion, 11 (16.7%) with abdominal malperfusion, and 7 (10.6%) patients with peripheral limb ischemia. According to the Penn classification (39) for the overall study cohort, 32 (48.5 %) patients were in class Aa, 21 (31.8%) were in class Ab, 6 (9%) patients were in class Ac, and 7 (10.6%) patients were in class Abc. There were no significant differences in terms of significant preoperative comorbidities and clinical presentation between the groups, A (<70 yrs old) and B (≥ 0 yrs old). The Penn classification did not differ between the two groups with 49 vs. 47% patients in class Aa, 34 vs. 26% in class Ab, 8.5 vs. 11% in class Ac, and 8.5 vs. 16% in class Abc (group A vs. group B respectively, *p* = 0.8). However, patients in group A were more frequently male (87 vs. 68%, *p* = 0.089) and had higher preoperative hemoglobin levels (13.1 ± 1.6 vs. 11.9 ± 1.8, *p* = 0.017). No deaths were recorded during the procedure. Operative characteristics and their distributions among the two groups are shown in Table 2. Concomitant procedures were needed in 32 (48.5%) patients: 11 (16.7%) coronary artery bypass grafting, 4 (6.0%) aortic valve replacement, 17 (25.7%) aortic root surgery, such as 12 (18.2%) Florida sleeve procedures and 5 (7.6%) modified Bentall. There was no difference between the two groups in terms of cardiopulmonary bypass time (205.1 ± 43.5 min in group A vs. 203 ± 50.3 min in group B, *P* = 0.7) and aortic cross-clamp time (118 ± 35.8 min in group A vs. 108 ± 36.9 min in group B, P = 0.2) However, the mean moderate hypothermic circulatory arrest time with a selective antegrade trivascular cerebral perfusion was significantly higher in group A (30 ±6.1 min in group A vs. 25.5 ± 7.9 min in group B, P = 0.046). Postoperative outcomes are summarized in Table 3. 30 days mortality was 10.6% for the entire cohort of patients (6.4% in group A vs 21% in group B, *P* = 0.1). Cumulative in-hospital mortality was 13.6% (8.5% in group A vs. 26% in group B, *P* = 0.11). No differences were found in terms of postoperative CVA (2.1% in group A vs. 10.5% in group B, *P* = 0.2), spinal cord injury (2.1% in group A vs. 5.3% in group B, *P* = 0.069) and AKI requiring hemodialysis (34% in group A vs. 21% in group B, P = 0.3). Also, the incidence of reopening for bleeding (10.6 vs. 5.3%, P=0.7), deep sternal wound infection (8.5 vs. 15.8%), respiratory failure requiring tracheostomy (25.5 vs 36.8%, P=0.4), and laryngeal nerve palsy (10.6 vs. 5.3%) did not differ among the two groups.

**Table 3:** Post-operative outcomes.

	**Overall (66)**	**Group A (47)**	**Group B (19)**	***p*-value**
30-days mortality	7 (10.6)	3 (6.4)	4 (21.0)	0.1
In-hospital mortality	9 (13.6)	4 (8.5)	5 (26.3)	0.11
ITU LOS (days)	15 (16.4)	15.3(16.9)	14.4 (15.4)	>0.9
In-hospital LOS (days)	27.7 (21.7)	27.5 (20.7)	28.3 (24.6)	0.78
Return to operating room	6 (9.1)	5 (10.6)	1 (5.3)	0.7
Prolonged ventilation	26 (39.3)	15 (31.9)	11 (57.9)	0.05
Respiratory failure	39 (59.1)	28 (59.6)	11 (57.9)	0.9
Tracheostomy	19 (28.8)	12 (25.5)	7 (36.8)	0.4
Pericardial effusion requiring drainage	15 (22.7)	11 (23.4)	4 (21.0)	>0.9
Pleural effusion requiring drainage	20 (30.3)	14 (29.8)	6 (31.6)	0.8
Deep sternal wound infection	7 (10.6)	4 (8.5)	3 (15.7)	0.4
Recurrent laryngeal nerve palsy	6 (9.1)	5 (10.6)	1 (5.3)	>0.9
AKI requiring CVVH	20 (30.3)	16 (34.0)	4 (21.0)	0.3
Spinal cord injury/paraplegia	2 (3.0)	1 (2.1)	1 (5.3)	0.069
Permanent CVA	3 (4.5)	1 (2.1)	2 (10.5)	0.2
Lower limb ischaemia	2 (3.0)	1 (2.1)	1 (5.3)	0.5

Group A: age at surgery <70 years; group B: age at surgery ≥ 70 years; ITU, Intensive Therapy Unit; LOS, Length of stay; CO, Cardiac Output; AKI, Acute Kidney Injury; CVVH, Continuous Veno-Venous Haemofiltration; CVA, Cerebrovascular Accident. Data are reported as mean (SD) for numerical variables and as count (%) for categorical variables.

**Figure 2:**
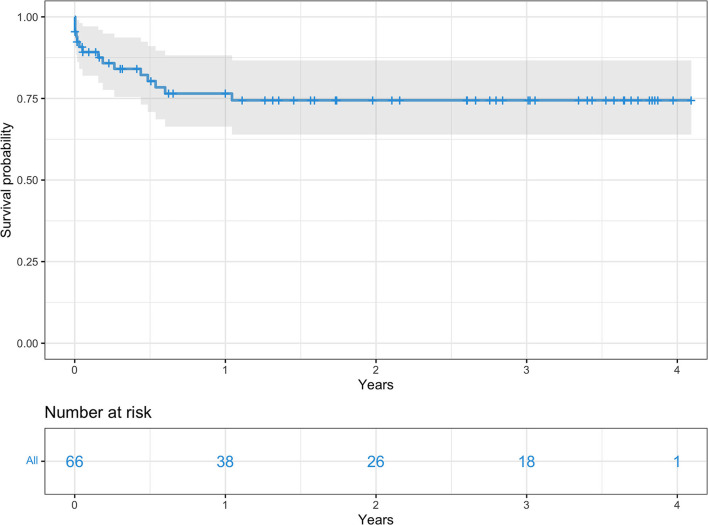
Kaplan-Meier survival curve for the overall surgical population.

**Figure 3:**
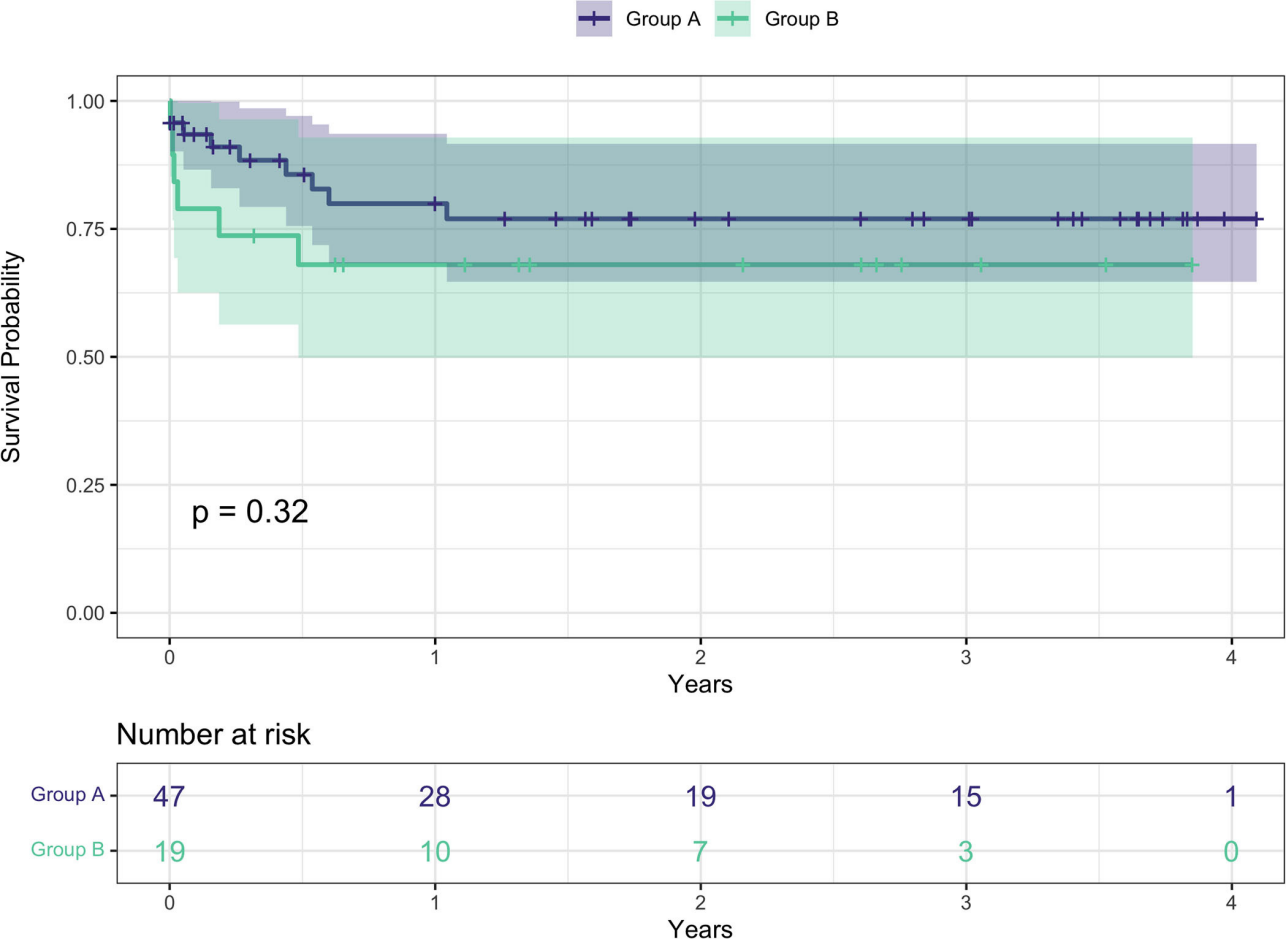
Kaplan-Meier survival curves between the two groups. Group A: age at surgery <70 years; group B: age at surgery ≥ 70.

Overall survival for the entire cohort at 3, 6, 12, and 24 months was 85, 80.3, 76.5, and 74.4% respectively (Figure 2). Survival rates by group were 91 vs. 73.7% at 3 months, 85.6 vs. 68% at 6 months, 79.9 vs. 68% at 12 months, and 77 vs. 68% at 24 months, all group A vs. group B, respectively (Figure 3).

The multivariable Cox proportional hazard model (Table 4) showed left ventricular ejection fraction (HR: 0.83, 95% CI: 0.79–0.92, *p* < 0.01), peripheral vascular disease (HR: 15.8, 95% CI: 3.9–62.9, *p* < 0.01), coronary malperfusion (HR: 0.10, 95% CI: 0.01-0.77, p =0.03), lower limbs malperfusion (HR: 5.1, 95% CI: 1.10–23.4, *p* = 0.04), and cardiopulmonary bypass time (HR: 1.02, 95% CI: 1 – 1.04, *p* = 0.01) as independent predictors of long term mortality.

**Table 4:** Multiple Cox PH model for long-term survival.

**Characteristics**	**HR**	**95% CI**	***P*-value**
Left ventricular ejection fraction	0.85	0.78–0.92	< 0.01
Peripheral vascular disease	15.8	3.9–62.9	< 0.01
Coronary malperfusion	0.10	0.01–0.77	0.03
Lower limb malperfusion	5.1	1.10–23.4	0.04
Cardiopulmonary bypass time	1.02	1.00–1.05	0.01

**Figure 4:**
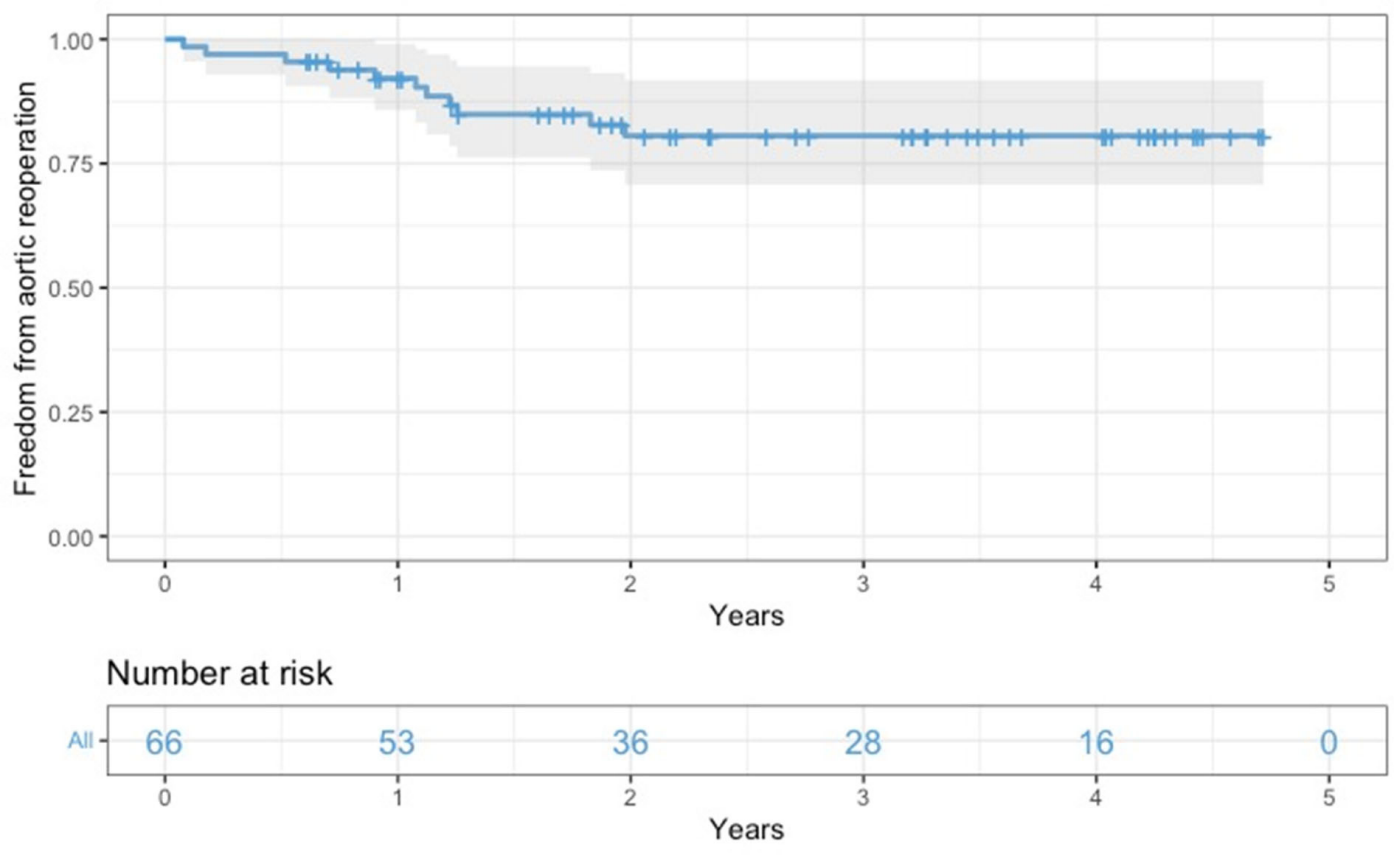
Kaplan-Meier freedom from aortic reoperations curve for the overall surgical population.

**Figure 5:**
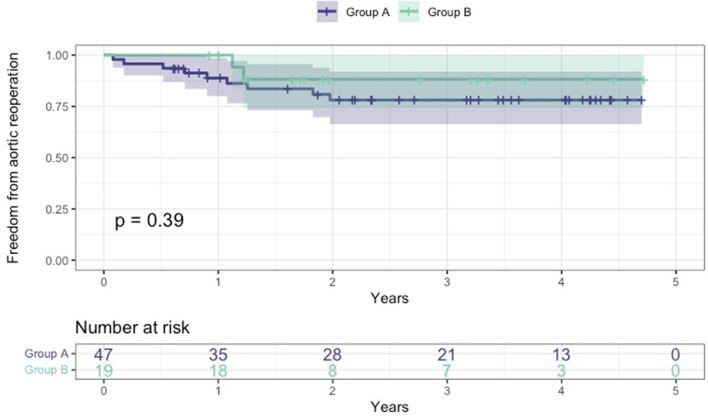
Kaplan-Meier freedom from aortic reoperation curves between the two groups. Group A: age at surgery <70 years; group B: age at surgery ≥70.

All survived patients underwent clinical and imaging follow-up until May 2022. According to our protocol, a postoperative CT angiogram was planned at 3, and 12 months after surgery and once yearly for the following 2 years. During this period, 11 patients required further aortic interventions: 10 patients (eight in group A and two in group B) underwent endovascular extension of the stent graft (TEVAR) using Relay Plus (Terumo Aortic, Scotland) covered endograft due to partial false lumen thrombosis/negative aortic remodeling as defined by Shesthra et al. (12). One patient developed a pseudoaneurysm at the level of proximal anastomosis which was successfully treated by positioning a vascular plug percutaneously under fluoroscopy *via* the right femoral artery. Freedom from aortic reinterventions for the entire cohort at 6, 12, and 24 months was 97, 92.2, and 80.6%, respectively (Figure 4); if considered by the group, freedom from aortic re-intervention rate was 97.9 vs. 100% at 6 months, 88.8 vs. 100% at 12 months and 78.1 vs. 88.2% at 24 months, all group A vs. group B, respectively (Figure 5).

## Discussion

There is an ongoing discussion about the most appropriate management of the aortic arch in the context of ATAAD, and particularly whether the FET technique should be consistently and systematically employed to treat these patients regardless of the primary involvement of the arch itself in the pathologic process. Our series proves that systematic use of the FET technique in an emergency setting to treat patients with ATAAD is feasible with good short-term and mid-term outcomes. In our study, 30-days and in-hospital mortality, CVA, and spinal cord injury were overall satisfactory and in line with the range reported in a contemporary review of literature recently published by our group where the FET technique was used as a surgical procedure in such complex setting (15). These results are even superior if compared to the data reported by recent reports of international registries where a more conservative approach with ascending aorta and hemiarch replacement was employed (3–5, 9).

Our experience with FET using Thoraflex hybrid in an emergency setting has been overall satisfactory; technical success was achieved in all patients. We did not pay attention to the physiological learning curve of this systematic approach in an acute setting with bad surgical results even in the first part of our experience. Correct cerebral perfusion during the hypothermic circulatory arrest is one of the main aspects determining neurological outcomes. As already reported, we believe that our peculiar “debranching first” strategy, as well as the trilateral selective antegrade cerebral perfusion during a moderate hypothermic circulatory arrest, is important to explain our particularly satisfactory results in terms of neurological complications, with a low overall incidence of permanent stroke (4.5%) compared to data from similar studies (36). This peculiar strategy allows uniform cerebral perfusion throughout the operation, except for the brief time needed for LCCA and LSA anastomosis, thus minimizing the cerebral ischemia time.

In our opinion, the claimed technical difficulties of this technique should not stop surgeons from adopting this surgical strategy in an acute setting, especially if a specialized centre with a high volume of aortic surgery is identified on a regional basis to receive and treat acute aortic syndrome (40–42). From a technical point of view, we believe that the FET hybrid prostheses, such as Thoraflex hybrid help to reduce the risk of aortic arch surgery in this context, by avoiding the need of performing a challenging distal anastomosis deeply at the level of the proximal DTA, where the risk of bleeding or rupture mainly due to the fragile dissected aortic wall is extremely high. Several authors, adopting systematically this strategy in an emergency setting, have stressed the importance of a hybrid prosthesis to face this challenging scenario (43).

Moreover, many patients affected by ATAAD present with distal aorta malperfusion due to the true lumen compression by the false lumen; in these cases, the FET technique can potentially re-expand the compressed true lumen and cover supplementary entry tears at the level of the proximal DTA, which may well sustain pressurization of the false lumen, thus reverting the malperfusion and improving surgical outcomes (44).

It is well-known that aortic dissection is a progressive disease and the natural history of the distal false lumen of the aorta following a primary procedure of ascending aorta or hemiarch replacement may lead to degeneration, aneurysm, and/or rupture, thus requiring additional extensive interventions. Data from large registries and recent literature suggest that in 60 to 90% of cases, a negative remodeling of the distal aorta takes place, to the extent of requiring second-stage endovascular or open surgical completion. In acute dissection, the FET technique can reduce DTA dilatation by bringing both coverages of secondary entry tears located in the proximal DTA and obliteration of the false lumen at the proximal DTA, thus reducing aortic-related deaths and the requirement for challenging distal aortic reinterventions (45–49). In our series, only 11 patients have required further aortic interventions: 10 patients underwent endovascular extension of the stent graft (TEVAR) using Relay Plus (Terumo Aortic, Scotland) covered endograft due to partial false lumen thrombosis, and 1 patient developed a pseudoaneurysm at the level of proximal anastomosis which was successfully treated by positioning a vascular plug percutaneously under fluoroscopy *via* the right femoral artery. This aspect supports the idea that the rate of aortic reintervention is lower if compared to conventional conservative treatment. Also, the rate of success for those patients was 100%, mainly due to less complex endovascular procedures when a hybrid vascular prosthesis has been implanted during the first surgical treatment.

In a recent paper, Beckmann et al. (50) have reported the results of a series of patients treated for non-urgent total aortic arch replacement using the FET technique. In their study worse perioperative mortality and morbidity as well as long-term survival was proved in septuagenarians than in younger patients. For this reason, to better clarify the impact of age on our cohort of patients, we have divided our study population into two groups: group A <70 years old and group B ≥70 years old. Our results showed no significant differences both for primary and secondary outcomes, thus supporting the thesis that in an acute setting, the age above 70 years old on its own does not impact negatively on perioperative outcomes and demonstrating that the use of FET in this setting is safe and effective even in the elderly population.

Although deeper evidence on long-term benefits is still needed, we believe that patients presenting with ATAAD should be at least considered for surgical treatment using the FET technique. Surely, the condition of the patient must be taken into account in deciding which approach should be adopted to treat this condition. In fact, even if the anatomy of the dissection is suitable, total arch replacement may be not indicated, such as in significantly elderly patients or in particularly poor clinical conditions with signs and symptoms of systemic malperfusion (18). In this regard, our subanalysis based on Penn classification showed that patients in class Ac and Abc, presenting with poor haemodynamic conditions, had worse outcomes if compared to patients in class Aa and/or Ab; particularly, 30 days and in-hospital mortality were significantly higher in class Ac and Abc (see Supplementary Table E1).

### Study limitations

The main limitation of this study is related to the retrospective and single-center nature of the study.

Moreover, the small number of patients included in this study represents a limitation in terms of statistical power and possible generalization of the results.

## Conclusion

Hybrid aortic arch and FET repair with the Thoraflex Hybrid graft to systematically treat emergency type A aortic dissection appears to be associated with good early- and mid-term clinical outcomes even in the elderly. Our perfusional strategy with a single pump and head vessel reimplantation as the last step has proved to offer highly effective cerebral protection while optimizing the heart and body ischemic time. Moreover, by inducing distal false lumen obliteration and thrombosis, the FET technique is likely to reduce the need for secondary procedures. Further studies to better clarify late complications are still needed.

## Author's note

Read as an e-poster (presentation on demand - director's choice) at American Association for Thoracic Surgery Aortic Symposium, Boston, USA, May 13-14, 2022.

## Data availability statement

The raw data supporting the conclusions of this article will be made available by the authors, without undue reservation.

## Ethics statement

The studies involving human participants were reviewed and approved by University Hospital San Giovanni di Dio e Ruggi d'Aragona. Written informed consent for participation was not required for this study in accordance with the national legislation and the institutional requirements.

## Author contributions

Conception and design: PC, GM, VB, PM, and SI. Administrative support: EM, VB, RC, MM, EM, and MD. Provision of study materials or patients: PC, RL, MM, MC, and FC. Collection and assembly of data: PC, GM, MM, MC, MD, DT, FC, and RC. Data analysis and interpretation: PC, VB, GM, PM, RC, FC, and SI. All authors contributed to the article and approved the submitted version.

## Funding

The authors declare that this study received funding from Serom Medical Technology S.r.l. The funder was not involved in the study design, collection, analysis, interpretation of data, the writing of this article, or the decision to submit it for publication.

## Conflict of interest

The authors declare that the research was conducted in the absence of any commercial or financial relationships that could be construed as a potential conflict of interest.

## Publisher's note

All claims expressed in this article are solely those of the authors and do not necessarily represent those of their affiliated organizations, or those of the publisher, the editors and the reviewers. Any product that may be evaluated in this article, or claim that may be made by its manufacturer, is not guaranteed or endorsed by the publisher.

## Abbreviations

AKI: acute kidney injury
ATAAD: acute type A aortic dissection
AVR: aortic valve replacement
CABG: coronary artery bypass grafting
CI: confidence interval
CKD: chronic kidney disease
COPD: chronic obstructive pulmonary disease
CPB: cardiopulmonary bypass
CVA: cerebro-vascular event
CVVH: continuous veno-venous haemofiltration
DSINEs: distal stent graft induced new entries
DTA: descending thoracic aorta
eGFR: estimated glomerular filtration rate
FET: frozen elephant trunk
HCA: hypothermic circulatory arrest
LCA: left common carotid artery
LOS: length of stay
LSA: left subclavian artery
SD: standard deviation
TEVAR: thoracic endovascular aortic repair.

## References

[1] El-Hamamsy I Ouzounian M Demers P McClure S Hassan A Dagenais F. Canadian thoracic aortic collaborative (CTAC). State-of-the-art surgical management of acute type A aortic dissection. Can J Cardiol. (2016) 32:100–9. 10.1016/j.cjca.2015.07.736 26604123

[2] Czerny M Schoenhoff F Etz C Englberger L Khaladj N Zierer A. The impact of pre-operative malperfusion on outcome in acute type a aortic dissection: results from the GERAADA registry. J Am Coll Cardiol. (2015) 65:2628–35. 10.1016/j.jacc.2015.04.030 26088302

[3] Berretta P Patel HJ Gleason TG Sundt TM Myrmel T Desai N. IRAD experience on surgical type A acute dissection patients: results and predictors of mortality. Ann Cardiothorac Surg. (2016) 5:346–51. 10.21037/acs.2016.05.10 27563547 PMC4973119

[4] Matsushita A Tabata M Fukui T Sato Y Matsuyama S Shimokawa T. Outcomes of contemporary emergency open surgery for type A acute aortic dissection in elderly patients. J Thorac Cardiovasc Surg. (2014) 147:290–4. 10.1016/j.jtcvs.2012.11 23228401

[5] Pape LA Awais M Woznicki EM Suzuki T Trimarchi S Evangelista A. Presentation, diagnosis, and outcomes of acute aortic dissection: 17-year trends from the international registry of acute aortic dissection. J Am Coll Cardiol. (2015) 66:350–8. 10.1016/j.jacc.2015.05.029 26205591

[6] Peterss S Pichlmaier M Curtis A Luehr M Born F Hagl C. Patient management in aortic arch surgery†. Eur J Cardiothorac Surg. (2017) 51:i4–i14. 10.1093/ejcts/ezw337 28108563

[7] Westaby S Saito S Katsumata T. Acute type A dissection: conservative methods provide consistently low mortality. Ann Thorac Surg. (2002) 73:707–13. 10.1016/S0003-4975(01)03449-X 11899170

[8] Kazui T Yamashita K Washiyama N Terada H Bashar AH Suzuki T Ohkura K. Impact of anaggressive surgical approach on surgical outcome in type A aortic dissection. Ann Thorac Surg. (2002) 74:S1844–7. 10.1016/s0003-4975(02)04155-3 12440678

[9] Di Bartolomeo R Pantaleo A Berretta P Murana G Castrovinci S Cefarelli M. Frozen elephant trunk surgery in acute aortic dissection. J Thorac Cardiovasc Surg. (2015) 149:S105–9. 10.1016/j.jtcvs.2014.07.098 25212056

[10] Shrestha M Fleissner F Ius F Koigeldiyev N Kaufeld T Beckmann E Martens A Haverich A. Total aortic arch replacement with frozen elephant trunk in acute type A aortic dissections: are we pushing the limits too far? Eur J Cardiothorac Surg. (2015) 47:361–6. 10.1093/ejcts/ezu185 24829403

[11] Watanuki H Ogino H Minatoya K Matsuda H Sasaki H Ando M. Is emergency total arch replacement with a modified elephant trunk technique justified for acute type A aortic dissection? Ann Thorac Surg. (2007) 84:1585–91. 10.1016/j.athoracsur.2007.06.045 17954066

[12] Shrestha M Bachet J Bavaria J Carrel TP De Paulis R Di Bartolomeo R. Current status and recommendations for use of the frozen elephant trunk technique: a position paper by the Vascular Domain of EACTS. Eur J Cardiothorac Surg. (2015) 47:759–69. 10.1093/ejcts/ezv085 25769463

[13] Rice RD Sandhu HK Leake SS Afifi RO. Is total arch replacement associated with worse outcomes during repair of acute type a aortic dissection? Ann Thorac Surg. (2015) 100:2159–65. 10.1016/j.athoracsur.2015.06.007 26271582

[14] Di Eusanio M Berretta P Cefarelli M Jacopo A Murana G Castrovinci S. Total arch replacement versus more conservative management in type A acute aortic dissection. Ann Thorac Surg. (2015) 100:88–94. 10.1016/j.athoracsur.2015.02.041 25979238

[15] Chivasso P Mastrogiovanni G Miele M Bruno VD Rosciano A Montella AP. Frozen elephant trunk technique in acute type A aortic dissection: is it for all? Medicina. (2021) 57:894. 10.3390/medicina57090894 34577818 PMC8467885

[16] Harky A Fok M Bashir M. Which is the optimal frozen elephant trunk? A systematic review and meta-analysis of outcomes in 2161 patients undergoing thoracic aortic aneurysm surgery using E-vita open plus hybrid stent graft vs. thoraflex hybrid prosthesis. Braz J Cardiovasc Surg. (2020) 35:427–36. 10.21470/1678-9741-2019-0220 32864920 PMC7454613

[17] Smith HN Boodhwani M Ouzounian M Saczkowski R Gregory AJ Herget EJ Appoo JJ. Classification and outcomes of extended arch repair for acute Type A aortic dissection: a systematic review and meta-analysis. Interact Cardiovasc Thorac Surg. (2017) 24:450–9. 10.1093/icvts/ivw355 28040765

[18] Berger T Czerny M. The frozen elephant trunk technique in acute and chronic aortic dissection: intraoperative setting and patient selection are key to success. Ann Cardiothorac Surg. (2020) 9:230–2. 10.21037/acs-2019-fet-10 32551258 PMC7298241

[19] Liu K Zhu C Zheng X Wang T Xu R Zhu Z. A new aortic arch inclusion technique with frozen elephant trunk for type A aortic dissection. Ann Surg. (2020) 271:978–83. 10.1097/SLA.0000000000003122 30531532

[20] Kaneyuki D Mogi K Watanabe H Otsu M Sakurai M Takahara Y. The frozen elephant trunk technique for acute retrograde type A aortic dissection: preliminary results. Interact Cardiovasc Thorac Surg. (2020) 31:813–9. 10.1093/icvts/ivaa199 33164059

[21] Inoue Y Matsuda H Omura A Seike Y Uehara K Sasaki H Kobayashi J. Comparative study of the frozen elephant trunk and classical elephant trunk techniques to supplement total arch replacement for acute type A aortic dissection. Eur J Cardiothorac Surg. (2019) 56:579–86. 10.1093/ejcts/ezz104 31005998

[22] Furutachi A Takamatsu M Nogami E Hamada K Yunoki J Itoh M Kamohara K. Early and mid-term outcomes of total arch replacement with the frozen elephant trunk technique for type A acute aortic dissection. Interact Cardiovasc Thorac Surg. (2019) 29:753–60. 10.1093/icvts/ivz154 31230069

[23] Katayama A Uchida N Katayama K Arakawa M Sueda T. The frozen elephant trunk technique for acute type a aortic dissection: results from 15 years of experience. Eur J Cardiothorac Surg. (2015) 47:355–60. 10.1093/ejcts/ezu173 24801338

[24] Xiao Z Meng W Zhu D Guo Y Zhang E. Treatment strategies for left subclavian artery during total arch replacement combined with stented elephant trunk implantation. J Thorac Cardiovasc Surg. (2014) 147:639–43. 10.1016/j.jtcvs.2013.02.013 23523037

[25] Yang SM Xu P Li CX Huang Q Gao HB Li ZF Chang Q. modified total arch replacement combined with a stented elephant trunk implantation for acute type A dissection under deep hypothermic circulatory arrest and selective antegrade cerebral perfusion. J Cardiothorac Surg. (2014) 9:140. 10.1186/s13019-014-0140-6 25174987 PMC4203861

[26] Shi E Gu T Yu Y Yu L Wang C Fang Q. Early and midterm outcomes of hemiarch replacement combined with stented elephant trunk in the management of acute DeBakey type I aortic dissection: comparison with total arch replacement. J Thorac Cardiovasc Surg. (2014) 148:2125–31. 10.1016/j.jtcvs.2013.10.058 24290707

[27] Ma WG Zheng J Dong SB Lu W Sun K Qi RD. Sun's procedure of total arch replacement using a tetrafurcated graft with stented elephant trunk implantation: analysis of early outcome in 398 patients with acute type A aortic dissection. Ann Cardiothorac Surg. (2013) 2:621–8. 10.3978/j.issn.2225-319X.2013.09.06 24109570 PMC3791189

[28] Hoffman A Damberg ALM Schälte G Mahnken AH Raweh A Autschbach R. Thoracic stent graft sizing for frozen elephant trunk repair in acute type A dissection. J Thorac Cardiovasc Surg. (2013) 145:964–9. 10.1016/j.jtcvs.2012.03.059 22507842

[29] Uchida N Katayama A Tamura K Sutoh M Kuraoka M Ishihara H. Frozen elephant trunk technique and partial remodelling for acute type a aortic dissection. Eur J Cardiothorac Surg. (2011) 40:1066–71. 10.1016/j.ejcts.2011.02.074 21511488

[30] Chen X Huang F Xu M Wang L Jiang Y Xiao L. The stented elephant trunk procedure combined total arch replacement for Debakey I aortic dissection: operative result and follow-up. Interact Cardiovasc Thorac Surg. (2010) 11:594–8. 10.1510/icvts.2010.238212 20716557

[31] Tsagakis K Pacini D Di Bartolomeo R Gorlitzer M Weiss G Grabenwoger M. Multicenter early experience with extended aortic repair in acute aortic dissection: is simultaneous descending stent grafting justified? J Thorac Cardiovasc Surg. (2010) 140(6 Suppl):S116–20. 10.1016/j.jtcvs.2010.07.066 21092776

[32] Pochettino A Brinkman WT Moeller P Szeto WY Moser W Cornelius K Bowen FW Woo YJ Bavaria JE. Antegrade thoracic stent grafting during repair of acute DeBakey I dissection prevents development of thoracoabdominal aortic aneurysms. Ann Thorac Surg. (2009) 88:482–9. 10.1016/j.athoracsur.2009.04.046 19632398

[33] Jakob H Tsagakis K Tossios P Massoudy P Thielmann M Buck T. Combining classic surgery with descending stent grafting for acute DeBakey type I dissection. Ann Thorac Surg. (2008) 86:95–101. 10.1016/j.athoracsur.2008.03.037 18573404

[34] Teasdale G Maas A Lecky F Manley G Stocchetti N Murray G. The Glasgow Coma Scale at 40 years: standing the test of time. Lancet Neurol. (2014) 13:844–54. 10.1016/S1474-4422(14)70120-6 25030516

[35] Furukawa T Uchida N Takahashi S Yamane Y Mochizuki S Yamada K. Management of cerebral malperfusion in surgical repair of acute type A aortic dissection. Eur J Cardiothorac Surg. (2017) 52:327–32. 10.1093/ejcts/ezx056 28369452

[36] Masiello P Mastrogiovanni G Presutto O Chivasso P Bruno VD Colombino M. Frozen elephant trunk procedure for complex aortic arch surgery: the Salerno experience with Thoraflex hybrid. J Card Surg. (2022) 37:107–14. 10.1111/jocs.16086 34662451 PMC9297964

[37] Masiello P Mastrogiovanni G Chivasso P Triggiani D Cafarelli F Iesu S. modified frozen elephant trunk hybrid device to facilitate supra-aortic trunk anastomosis. J Card Surg. (2021) 36:371–3. 10.1111/jocs.15208 33225461

[38] R Core Team. R: A Language and Environment for Statistical Computing. Vienna, Austria: R Foundation for Statistical Computing (2019). Available online at: https://www.Rproject.org/

[39] Augoustides JG Geirsson A Szeto WY Walsh EK Cornelius B Pochettino A. Observational study of mortality risk stratification by ischemic presentation in patients with acute type A aortic dissection: the Penn classification. Nat Clin Pract Cardiovasc Med. (2009) 6:140–6. 10.1038/ncpcardio1417 19065126

[40] Andersen ND Ganapathi AM Hanna JM Williams JB Gaca JG Hughes GC. Outcomes of acute type a dissection repair before and after implementation of a multidisciplinary thoracic aortic surgery program. J Am Coll Cardiol. (2014) 63:1796–803. 10.1016/j.jacc.2013.10.085 24412454 PMC4159705

[41] Mariscalco G Maselli D Zanobini M Ahmed A Bruno VD Benedetto U Gherli R Gherli T Nicolini F. Aortic centres should represent the standard of care for acute aortic syndrome. Eur J Prev Cardiol. (2018) 25(1_suppl):3–14. 10.1177/2047487318764963 29708034

[42] Chikwe J Cavallaro P Itagaki S Seigerman M Diluozzo G Adams DH. National outcomes in acute aortic dissection: influence of surgeon and institutional volume on operative mortality. Ann Thorac Surg. (2013) 95:1563–9. 10.1016/j.athoracsur.2013.02.039 23562465

[43] Di Eusanio M Petridis FD Pacini D Di Bartolomeo R. Facilitated aortic arch repair with the frozen elephant trunk technique. Eur J Cardiothorac Surg. (2011) 40:1261–2. 10.1016/j.ejcts.2011.02.017 21435896

[44] Di Marco L Pantaleo A Leone A Murana G Di Bartolomeo R Pacini D. The frozen elephant trunk technique: european association for cardio-thoracic surgery position and bologna experience. Korean J Thorac Cardiovasc Surg. (2017) 50:1–7. 10.5090/kjtcs.2017.50.1.1 28180096 PMC5295476

[45] Kimura N Itoh S Yuri K Adachi K Matsumoto H Yamaguchi A Adachi H. Reoperation for enlargement of the distal aorta after initial surgery for acute type A aortic dissection. J Thorac Cardiovasc Surg. (2015) 149(2 Suppl):S91-8.e1. 10.1016/j.jtcvs.2014.08.008 25224548

[46] Geirsson A Bavaria JE Swarr D Keane MG Woo YJ Szeto WY Pochettino A. Fate of the residual distal and proximal aorta after acute type a dissection repair using a contemporary surgical reconstruction algorithm. Ann Thorac Surg. (2007) 84:1955–64. 10.1016/j.athoracsur.2007.07.017 18036916

[47] Fattouch K Sampognaro R Navarra E Caruso M Pisano C Coppola G. Long-term results after repair of type a acute aortic dissection according to false lumen patency. Ann Thorac Surg. (2009) 88:1244–50. 10.1016/j.athoracsur.2009.06.055 19766814

[48] Evangelista A Salas A Ribera A Ferreira-González I Cuellar H Pineda V. Long-term outcome of aortic dissection with patent false lumen: predictive role of entry tear size and location. Circulation. (2012) 125:3133–41. 10.1161/CIRCULATIONAHA.111.090266 22615344

[49] Tsagakis K Dohle DS Jánosi RA. Abstract 19090: Identification and classification of descending aorta re-entry sites in type I aortic dissection. Circulation. (2015) 132:S3. 10.1161/circ.132.suppl_3.1909026078378

[50] Beckmann E Martens A Kaufeld T Natanov R Krueger H Haverich A. Is total aortic arch replacement with the frozen elephant trunk procedure reasonable in elderly patients? Eur J Cardiothorac Surg. (2021) 60:131–7. 10.1093/ejcts/ezab063 33582774

