# Supplementary material for: Systematic total arch replacement with thoraflex hybrid graft in acute type A aortic dissection: A single centre experience
Source: Front Cardiovasc Med. 2022 Oct 14;9:997961. doi: 10.3389/fcvm.2022.997961 (PMC9614841; doi:10.3389/fcvm.2022.997961)
Supplement: Supplementary file 1 [file Table_1.pdf]

Table E1. Postoperative outcomes

	Penn Aa (n. 32)	Penn Ab (n. 21)	Penn Ac (n. 6)	Penn Abc (n. 7)	p value
30-days mortality	0 (0)	2 (9.5)	2 (33)	3 (43)	0.001
In-hospital mortality	1 (3.1)	2 (9.5)	3 (50)	3 (43)	0.002
Return to operating room	3 (9.4)	1 (4.8)	1 (17%)	1 (14)	0.5
Low CO syndrome	9 (28)	8 (38)	3 (50)	4 (57)	0.4
Prolonged ventilation	9 (28)	11 (52)	2 (33)	4 (57)	0.2
Respiratory failure	17 (53)	14 (67)	4 (67)	4 (57)	0.8
Tracheostomy	7 (22)	6 (29)	3 (50)	3 (43)	0.4
Pericardial effusion requiring drainage	12(38)	2 (9.5)	0 (0)	1 (17)	0.059
Pleural effusion requiring drainage	9 (28)	6 (29)	1 (17)	4 (67)	0.3
Deep sternal wound infection	4 (12)	0 (0)	2 (33)	1 (14)	0.068
Recurrent laryngeal nerve palsy	4 (12)	1 (4.8)	1 (17)	0 (0)	0.7
AKI requiring CVVH	7 (22)	7 (33)	1 (17)	5 (71)	0.073
Spinal cord injury/paraplegia	0 (0)	1 (4.8)	1 (17)	0 (0)	0.054
Permanent CVA	2 (6.2)	1 (4.8)	0 (0)	0 (0)	>0.9
Lower limb ischaemia	1 (3.1)	1 (4.8)	0 (0)	0 (0)	>0.9

Group A: age at surgery <70 years; group B: age at surgery ≥ 70 years; ITU, Intensive Therapy Unit; LOS, Lenght of stay; CO, Cardiac Output; AKI, Acute Kidney Injury; CVVH, Continuous Veno-Venous Haemofiltartion; CVA, Cerebro-Vascular Accident. Data are reported as mean (SD) for numerical variables and as count (%) for categorical variables.
